# GHB–Induced Cognitive Deficits During Adolescence and the Role of NMDA Receptor

**DOI:** 10.2174/157015911795017038

**Published:** 2011-03

**Authors:** R Sircar, L-C Wu, K Reddy, D Sircar, A.K Basak

**Affiliations:** 1 The Feinstein Institute for Medical Research, Bronx, Neurology, PathologyAlbert Einstein College of Medicine, Bronx, NY, USA; 2 Departments of Psychiatry & Behavioral Sciences, Neurology, PathologyAlbert Einstein College of Medicine, Bronx, NY, USA

**Keywords:** Addiction, juvenile, cognitive function, glutamatergic, radioligand binding, western blot, NR subunit, GHB.

## Abstract

We have earlier reported that γ-hydroxybutyric acid (GHB) disrupts the acquisition of spatial learning and memory in adolescent rats. GHB is known to interact with several neurotransmitter systems that have been implicated in cognitive functioning. The *N*-methyl-D-aspartate receptor (NR) -type of glutamate receptor is considered to be an important target for spatial learning and memory. Molecular mechanisms governing the neuroadptations following repeated GHB treatment in adolecent rats remain unknown. We examined the role of NMDA receptor in adolescent GHB-induced cognitive deficit. Adolescent rats were administered with GHB on 6 consecutive days, and surface-expressed NMDA receptor subunits levels were measured. GHB significantly decreased NR1 levels in the frontal cortex. Adolescent GHB also significantly reduced cortical NR2A subunit levels. Our findings support the hypothesis that adolescent GHB-induced cogntive deficits are associated with neuroadaptations in glutamatergic transmission, particulaly NR functioning in the frontal cortex.

## 1. INTRODUCTION

γ-hydroxybutyric acid (GHB), a short-chain fatty acid found endogenously in the brain [1], rapidly crosses the blood brain barrier following systemic administration resulting in CNS mediated effects. GHB is known to induce short-term anterograde amnesia in humans, [2-4], and this GHB-induced amnesia is thought to play a prominent role in drug-facilitated sexual assault [4-6]. Although GHB-induced memory impairments have been reported in humans relatively few studies have looked at the effects of GHB on learning and memory in animals. In monkeys, GHB failed to have any effect on the go/no-go visual discrimination task [7]. Navarro and coworkers studied the effects of chronic low doses of GHB (5-100 mg/kg for 12 - 30 days) on the “hole-board” task performance in adult mice, and reported that working memory was significantly reduced in GHB-treated mice compared to controls [8-10]. In adult rats, GHB has been shown to decrease operant behavior but not working memory [11]. Earlier, our laboratory has shown that repeated GHB exposure in adolescent rats significantly impaired the acquisition of spatial learning and memory [12].

The precise mechanisms underlying GHB actions remain unclear, but evidence supports that GHB acts *via* several different receptors. Since subcellularly GHB is located within the presynaptic terminal, it is thought to act as a neurotransmitter/neuromodulator [13-15]. GHB is known to bind with specific brain receptors that have a distinct anatomical distribution pattern with high densities of [^3^H]GHB binding sites in the hippocampus including dentate gyrus, frontal cortex, septum, nucleus accumbens and caudate-putamen. Areas such as the cerebellum, hypothalamus, and pons-medulla are almost devoid of any such binding [13-17]. GHB has also been shown to interact with the GABA_B_ receptor [18].

Glutamate signalling is thought to play a significant regulatory role in neurocognitive functioning. GHB has been shown to increase glutamate release, and GHB-induced glutamate release can be blocked by NR antagonists [19, 20]. GHB–induced seizure can be suppressed by NMDA antagonists [21]. Pretreatment with competitive (CGP 43487) and non-competitive (MK-801 and ketamine) NMDA receptor antagonist dose dependently suppressed GHB-induced withdrawal seizures. Since spatial learning and memory have been associated with the NMDA receptor, and GHB impairs the acquisition of this type of memory, we tested the hypothesis that GHB-induced memory deficit is associated with alterations in NMDA receptor functioning.

## 2. MATERIALS AND METHODS

### 2.1. Animals

Adolescent male Sprague-Dawley rats (Taconic, Germantown, MD), were used. Rats were housed in our temperature- and humidity-controlled animal care facility, with a 12 hr light\12 hr dark cycle and at a room temperature of 22°C. They were kept in plastic cages, three to a cage, with *ad libitum* access to food and water. Each rat was allowed a minimum of 2-4 days to habituate to the colony before testing began. They were briefly handled each day during this habituation process. All experimental protocols used were approved by the institutional Animal Review Committee, and were in compliance with the NIH Guide for the Care and Use of Laboratory Animals.

### 2.2. Drug Treatment

Rats were randomly assigned to one of two groups – saline, or GHB (100 mg/kg). All treatments were administered daily by intraperitoneal (ip) injections for 6 consecutive days. Controls rats received isovolumetric ip saline injections at the same time. Fresh solutions of GHB were prepared in physiological saline for each batch of rats. Drug solutions were refrigerated after injections, and warmed to room temperature prior to injections. Rats were sacrificed, and their brains removed. Frontal cortex was dissected and rapidly frozen on dry ice, and kept at -80°C until use. Different sets of animals were used to measure the effects of repeated GHB treatment on NR binding and on NR subunit protein expression. 

### 2.3. [^3^H] MK-801 Binding

Experimental protocols for synaptosomal membrane preparation and [^3^H]MK-801 binding were as described before [12, 22]. On the day of membrane preparation, tissue samples were homogenized in 50 mM Tris-HCl and the homogenate was centrifuged for 15 min at 4°C. The pellet was suspended in Triton X-100 (0.04%) and incubated in a 37°C water bath for 20 min. Tris-HCl (100 mM) was added to the suspension, and centrifuged for 20 min. The supernatant was discarded and the pellet was resuspended in 50 mM Tris-HCl. The homogenate was centrifuged and the pellet was washed three times. After the last spin the pellet was suspended in 6 vol 0.32 M sucrose and stored at –80°C till needed. On the day of the binding experiment, the frozen Triton prep was thawed on ice and centrifuged for 15 min. The pellet was resuspended in 6 vol 5 mM Tris-acetate buffer and centrifuged for 20 min. The pellet was washed three times. The final pellet was suspended in Tris-acetate buffer. Two hundred and fifty µl of the membrane suspension was incubated with graded concentrations of [^3^H]MK-801 (0.5–30 nM) for 90 min, in the presence of 100 µM L-glutamate and 100 µM glycine. Binding reaction was terminated by rapid filtration under vacuum through GF/B filters presoaked in 0.1% polyethyleneimine. Filters were washed twice and radioactivity was measured by spectrophotometry. Protein concentrations in samples of membrane suspension were determined according to Peterson’s modification of Lowry’s method using the Sigma Protein Kit (Sigma, St. Louis, MO). 

### 2.4. Western Blotting for NR Proteins 

Frozen brain regions were thawed, lysed in radioimmunoprecipitation assay (RIPA) buffer (1X PBS, 1% NP-40, 0.5% sodium deoxycholate, 0.1% SDS) containing protease inhibitor, and homogenized for 10 seconds on ice. The homogenate was transferred to an eppendorf tube and 0.1M PMSF (serine protease inhibitor) was added. The suspension was incubated on ice for 20 min, sonicated for 10 sec and then spun at 15,000xg for 10 min at 4°C. Supernatant was carefully collected without disturbing the pellet; pellet contained nuclei and was discarded. Protein concentration in tissue lysate was measured using commercially available protein estimation kit (Pierce BCA Protein Assay kit, Thermo Fisher Scientific, Rockford, IL). Equal amounts of protein (40 µg) from GHB-treated and control frontal cortices were separated on 7.5% (w/v) SDS-PAGE electrophoresis. Following electrophoretic transfer of proteins onto nitrocellulose membrane (Trans Blot, Bio-Rad, Hercules, CA), membranes were incubated overnight with the primary monoclonal antibody (NR1, NR2A, NR2B; 1:3000; BD Biosciences, San Jose, CA) at 4°C. NR1 antibody detected all the NR1 splice variants. Blots were washed four times for 5 min with the wash buffer, and then incubated with the secondary antibody (goat anti-mouse, Santa Cruz Biotechnology, Santa Cruz, CA) diluted in wash buffer containing 5% (w/v) nonfat milk. Immunoreactive bands were visualized using ECL detection system (Perkin-Elmer, Waltham MA). Molecular weights were estimated using prestained protein standards (Rainbow molecular weight standard, Amersham). 

Quantitation of western blots: To control for loading efficiency, blots were stripped and reprobed with β-actin (Sigma). The immunoreactive bands were scanned and quantified using a densitometric system (Bio-Rad GS-800 scanner) and analyzed (BioRad Quantity One® Analysis Software, Hercules, CA). The densitometric volume for each band was calculated as the product of intensity (expressed in pixels) for the area of the band. The density values of the immunoreactive NR subunit bands were divided by the density values of the immunoreactive β-actin bands from the corresponding gel lanes. Background correction values were subtracted from each lane to minimize the variability across membranes.

### 2.5. Data Analysis and Statistics

For both radioligand binding and western blot analysis, 3-6 independent experiments were performed. Average values from these experiments are reported as mean ± sem. Saturation binding experiments were analyzed using the Prizm^®^ curve-fitting and receptor binding analysis program (GraphPad, San Diego, CA). Saturation binding experiments were analyzed using the Prism^®^ curve-fitting and receptor binding analysis program (GraphPad, San Diego, CA). Statistical comparisons between each treatment group were carried out using the student *t* test (p< 0.05). All statistical comparisons were performed using the Prism® software (GraphPad, San Diego, CA).

## 3. RESULTS

### 3.1. Adolescent GHB Administration on [^3^H]MK-801 Binding

Saturation [^3^H]MK-801 binding experiments were carried out in the frontal cortex of GHB-treated rats and saline controls. Scatchard plots were created to determine whether treatment effects involved changes in receptor affinity (*K_d_*) and/or maximal binding. Under the binding conditions used a single binding site was detected. There were no differences in the *K_d_* values between control and drug-treated brains. GHB treatment decreased [^3^H]MK-801 binding to the NMDA receptor-channel complex. 

### 3.2. Adolescent GHB Administration on NR Subunit Levels

**Fig. (1):**
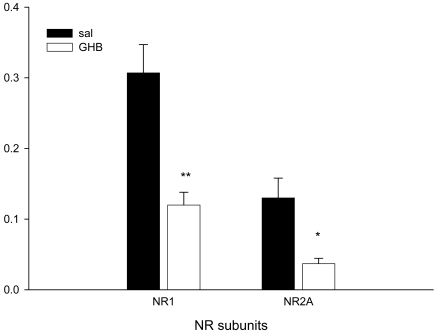
Repeated GHB administration alters the expression of the NR1 and NR2B receptor subunit proteins. NMDA receptor subunit protein expression was normalized to the apparent levels of b-actin in each sample. Compared to saline-treated controls, GHB decreased both NR1- and NR2A-like immunoreactivities in the frontal cortex. **p<0.01; *p<0.05

Changes in NMDA receptor subunit proteins following exposure to GHB were examined. To minimize the influence of sample-to-sample differences in quality and to control for errors in quantification or sample loading, NR1, NR2A and NR2B subunit levels were normalized to β-actin expression in each sample. GHB treatment did not significantly influence β-actin-like immunoreactivity across all experiments. Both β-actin-normalized NR1 and NR2A  subunit levels were significantly lower in GHB-treated brain compared to saline-treated controls (Fig **1**). Normalized NR2B subunit levels showed a tendency to increase in GHB-treated rats compared to saline-treated rats (1.05 ± 0.27 and 0.404 ± 0.11, respectively), but the difference was not statistically significant.

## DISCUSSION

Earlier we have reported that GHB impairs spatial learning and memory [12]. Here we report that GHB-induced memory impairments are associated with adaptive alterations in the excitatory glutamatergic system mediated by the NMDA receptor-gated ion channel. Repeated GHB administration decreased the maximal density of NMDA binding sites. There was a concomitant reduction in the levels of NR1 subunit as determined from the western blot studies. Levels of the NR2A subunit were also significantly lower in the frontal cortex of GHB administered rats compared to saline-treated controls. NR2B subunit levels appeared to be higher in GHB-treated rats than the saline-treated rats but the effect was not statistically significant. These findings suggest that some of the neuroadaptative changes following repeated GHB exposure are mediated by the NMDA receptor.

NMDA receptor is known to be crucial for mnemonic processes [23]. Repeated GHB administrations in adolescent rats resulted in decreased NR receptor binding along with a reduction in the levels of NR1 subunit, the constitutive NMDA receptor subunit. GHB has been reported to increase glutamate levels in the brain [19]. Increased excitatory amino acid levels in the brain following GHB exposure may be responsible for the down regulation of the NMDA receptor. Reduction in NR functioning as measured by [^3^H]MK-801 binding, has been reported for other drugs of abuse. Rats self administrating cocaine for seven days show significantly lower levels of NMDA receptor binding in the medial prefrontal cortex compared to saline-treated controls [2, 24]. 

Functional NMDA receptors are composed of the obligatory NR1 subunit along with one or more members of the NR2 and/or NR3 subunit families. The presence of specific NR2 subunits significantly influences the pharmacological characteristics of the NMDA receptor. Since [^3^H]MK-801, a functional marker of NMDA channel, was lower in GHB-treated frontal cortex compared to saline controls, it is not surprising that the constitutive NR1 subunit levels were also lower in the frontal cortex of GHB-treated rats than saline-treated rats. Rats treated with methamphetamine, another abused substance, have been shown to have lower NMDA receptor functioning as reflected by significant reductions in NR1 subunit expression in the frontal cortex [25]. 

It is interesting that the NR2A subunit protein levels were altered by repeated GHB exposure. NR2A subunit is thought to be important in synaptic plasticity, and in learning and memory. Mice lacking in NR2A receptor subunit (also referred to as GluRε1) exhibit reduced long tem potentiation (LTP), as well as impairments in spatial learning and memory [26, 27]. NR2A subunit levels are reduced in animals exposed to lead during development, and this is associated with impairments in LTP [7]. 

Alterations in NR2B levels following adolescent GHB exposure did not reach statistical significance. Among the various NR subunits, the NR2B subunit expression is highly regulated during brain development. Normally it is the NR2B subunit that appears to be sensitive to drug exposure. One of the adaptive changes consistently seen following ethanol treatment is an upregulation of the NR2B subunit protein [28, 29].

Together, the present study suggests that GHB exposure during adolescence results in adaptive regulations of the NMDA receptor system. Repeated GHB downregulates NR levels in the frontal cortex, and changes the heteromeric combination of the NMDA receptor subunits. Reduction in NR1/NR2A assembly appears to be sensitive to inhibition by GHB, and provides a mechanistic link to GHB-induced impairments in spatial leaning and memory.
